# Neebe’s Treatment of Sweating Feet by Crude Hydrochloric Acid

**Published:** 1894-09

**Authors:** 


NEEBE'S TREATMENT OF SWEATING FEET BY CRUDE HYDROCHLORIC ACID.—When the feet are very tender, especially in hot weather, treatment is preceded by an eight to ten days' application of compound talc powder, which is sprinkled in the stockings. The application of the acid is best made in the evening. The crude hydrochloric acid is poured into a flat vessel of stone or glass or porcelain, sufficiently large to receive the two feet. Since the soles of the feet and the skin between the toes are the seat of the trouble, sufficient hydrochloric acid is poured into the vessel to completely cover the soles. It should not be allowed to come in contact with the skin of the back of the feet. The heel is kept in the acid for five minutes; then the sole of the foot for ten menutes. After this the feet, especially the skin between the toes, are washed in soap and warm water. Soaking in the acid must be at once stopped as soon as pain is excited, and the painful spots must be treated with zinc ointment until healing is complete. The process of soaking may be repeated twice weekly, and continued for from five to eight weeks in obstinate cases.—*Med. Press and Circular.*

